# Can the Entire Function of the Foot Be Concentrated in the Forefoot Area during the Running Stance Phase? A Finite Element Study of Different Shoe Soles

**DOI:** 10.5114/jhk/174311

**Published:** 2023-11-28

**Authors:** Huiyu Zhou, Datao Xu, Wenjing Quan, Ukadike Chris Ugbolue, Zhanyi Zhou, Yaodong Gu

**Affiliations:** 1 Faculty of Sports Science, Ningbo University, Ningbo, China.; 2 School of Health and Life Sciences, University of the West of Scotland, Scotland, UK.; 3 Faculty of Engineering, University of Pannonia, Veszprem, Hungary.; 4 Savaria Institute of Technology, Eotvos Lorand University, Budapest, Hungary.; 5 Department of Radiology, Ningbo No. 2 Hospital, Ningbo, China.

**Keywords:** shoes, unstable conditions, feature classification and recognition

## Abstract

The goal of this study was to use the finite element (FE) method to compare and study the differences between bionic shoes (BS) and normal shoes (NS) forefoot strike patterns when running. In addition, we separated the forefoot area when forefoot running as a way to create a small and independent area of instability. An adult male of Chinese descent was recruited for this investigation (age: 26 years old; body height: 185 cm; body mass: 82 kg) (forefoot strike patterns). We analyzed forefoot running under two different conditions through FE analysis, and used bone stress distribution feature classification and recognition for further analysis. The metatarsal stress values in forefoot strike patterns with BS were less than with NS. Additionally, the bone stress classification of features and the recognition accuracy rate of metatarsal (MT) 2, MT3 and MT5 were higher than other foot bones in the first 5%, 10%, 20% and 50% of nodes. BS forefoot running helped reduce the probability of occurrence of metatarsal stress fractures. In addition, the findings further revealed that BS may have important implications for the prevention of hallux valgus, which may be more effective in adolescent children. Finally, this study presents a post-processing method for FE results, which is of great significance for further understanding and exploration of FE results.

## Introduction

The primary purpose of daily shoes is to preserve and enhance the stability and postural control of the human body, particularly the foot, during athletics (Reinschmidt and Nigg, 2000). As a result of the ongoing advancements in footwear research and development, sports shoes were developed in the 1970s with a variety of goals in mind, including enhancing athletic performance and lowering sports injuries. The necessity for footwear progressively changed from conventional uses like foot protection and shock absorption to demands specific to various sports in order to fulfill a variety of roles. In response to customer demand, contemporary businesses and marketplaces are concentrating their design ideas on shock absorption, motion control, and adjustment of plantar pressure distribution in an effort to provide training equipment that enhances performance while lowering the risk of injury (Nigg and Enders, 2013). It is from this idea that “unstable construction footwear” is conceived.

**Figure 1:**
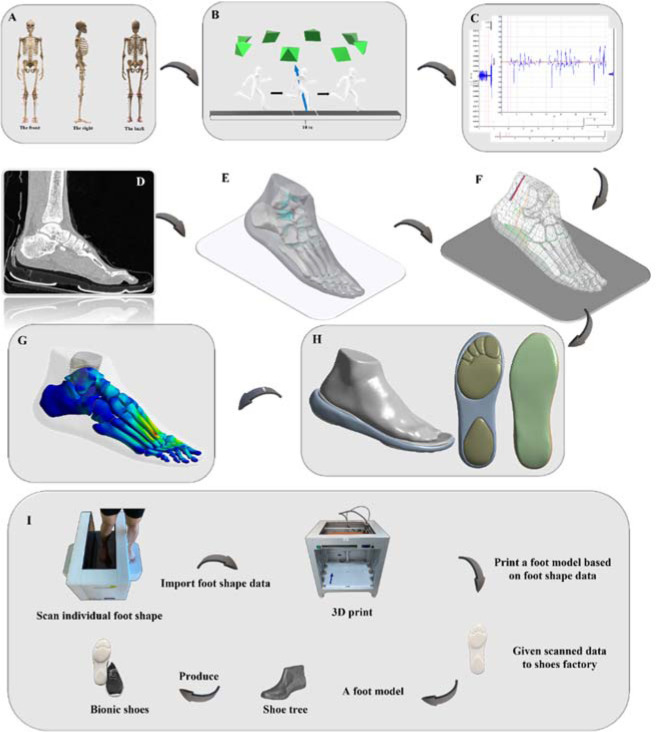
Pictorial illustration of the biomechanical steps applied to the investigation. A: Display of retroreflective markers placed on lower limb joints and segments; B: Description of the experiment setup designed to gather data on dynamics and kinematics; C: Outcomes of the muscular force; D: CT and MRI scans; E: Illustration of bones and cartilages; F: Illustration of muscles and ligaments; H: Illustration showing the two distinct types of shoes; G: Illustration of the simulation's ultimate output; I: Representation figure depicting the steps involved in creating BS.

It is compatible with ideas of human evolution because evidence points to barefoot walking being frequent among early people (Richmond et al., 2010). Several people in the world's indigenous cultures still walk or run barefooted. This suggests that footwear is not seen as crucial to human existence or given high importance. With this in mind, we designed BS (Figure 1) to combine the benefits of being barefoot with the security of wearing shoes. With respect to other forms of unstable footwear, bionic shoes (BS) were designed to be off-balance only when the wearer changes their posture or movements. As mentioned in previous studies, the most critical factor affecting stability is not the difference in posture, but probably more because of the difference in unstable structures (Hicks, 1953). Instead, our BS combine these benefits and integrate the barefoot form, more accurately mimicking and reproducing the experience of running and walking barefoot.

Based on the findings of biomechanical kinematic research on MBT (Masai Barefoot Technology) shoes (Nigg et al., 2012), this study indicates a notable increase in the sagittal plane joint angle or range of motion during walking when comparing MBT shoes to conventional footwear. This effect is observed in the ankle, knee, and hip joints. This observation clarifies that the rounded configuration of the MBT sole in the anterior-posterior direction necessitates an alteration in the joint angle during forward locomotion, thereby affecting the range of motion. One potential advantage is that an increased joint angle can enhance the cushioning effect on the lower limb joints (Bennett et al., 2009), thereby reducing the impact experienced by these joints. On the contrary, when considering the perspective of kinematics, it is important to note that the applicability of MBT shoes is restricted to specific scenarios or individuals, rather than being generally suitable for every person. In contrast, the frontal plane is responsible for the most notable alterations in the kinematics of the lower limbs during walking and running (Gu et al., 2014; Mei et al., 2015; Mitschke et al., 2019). This is due to its resemblance to the structure of a barefoot and its consequential influence on biomechanical outcomes. By realistically restoring the instability when barefoot, the role of improving the stability of the lower limb and foot function is enhanced. It is noteworthy to mention that certain researchers have obtained unexpected findings in relation to the single-leg landing test. These findings suggest that the utilization of BS can effectively enhance the flexion angle of the knee and hip, thereby improving the cushioning mechanism during landing. Zhou et al. (2018) stated that the observed phenomenon could be attributed to the organism's ability to sense the inherent instability during the preparation phase, leading to a condition comparable to pre-activation. This finding provides evidence that the medial-lateral instability shoe has an impact on sagittal motion data to some degree, and this alteration in the joint angle is undeniably advantageous.

With its 26 bones, 33 joints, and plethora of muscles, tendons, ligaments, cartilage, and other tissues, a healthy human foot is an incredibly robust mechanical structure. The foot serves as the first point of contact between the human body's internal kinetic chain and the external movement environment during static standing, as well as during dynamic running and jumping (Hicks, 1954). The foot can be divided into the heel (talus and calcaneus), the middle part of the foot (navicular, cuboid and cuneiform bones), and the forefoot (metatarsal and phalanx bones). Different regions have different functions, therefore, many studies on the foot have analyzed plantar pressure by dividing it into different plantar divisions (Orlin and McPoil, 2000). On the contrary, the current research on unstable condition shoes agrees that the sole should be a monolithic structure (Nigg et al., 2006). But then the question does arise as to why the unstable sole structure is a monolithic structure at the time of the design, considering that the foot is divided into three sections and there are multiple areas to be explored during the analysis.

Normal biomechanical testing procedures do not accurately represent the foot state of change. It seems that the finite element (FE) analysis technique is the best way to demonstrate the mechanical responses of biological systems under complex loading circumstances (Gefen et al., 2000). FE analysis may be used to anticipate internal stresses and strains, as well as the distribution of loads throughout the foot's different components. Moreover, it enables parametric evaluations for the sole design and material research, both of which are important for anticipating the foot function (Gu and Li, 2005; Gu et al., 2010). However, when comparing the stress characteristics of various models after the FE analysis, there are a few limitations to this method that should be addressed (Szabó and Babuška, 2021; Zhang et al., 2023). To put it another way, this comparison following FE analysis is typically based on stress distribution trends and maximum stress values (Zhang et al., 2023), which is a specific contingency (Xiang et al., 2022). Previous research has utilized the F-test technique to investigate the stress levels at each bone node (Szabó and Babuška, 2021). While this approach is useful for preventing the occurrence of maximum stress value, it does not take into account the useful data regarding the characteristics of the stress distribution. Therefore, in biomechanics, it has become difficult to analyze the stress distribution characteristics of bones in post-processing of FE without increasing the risk of the existence of stress extremes (Gu et al., 2010; Xu et al., 2022b).

The goal of this study was to use the FE method to compare and study the differences between BS and NS forefoot strike patterns when running. In addition, we separated the forefoot area when forefoot running as a way to create a small and independent area of instability. We hypothesized that the foot bone stress value would be lower when running on the forefoot with BS than when running with NS. More specifically, we further hypothesized that the metatarsal bones stress value would be lower when running on the forefoot with BS than when running with NS.

## Methods

### Workflow

This investigation can be described as composed of three parts (Figure 1 A-H): First, to determine the muscle force, we imported the data obtained from the Vicon Motion System (Oxford Metrics Ltd., Oxford, UK) into the simulation software OpenSim (Stanford University, Stanford, CA, USA). Second, we obtained foot MRI and CT pictures, then uploaded the layer scans into the modeling program. The image quality was improved by applying the smooth function and altering the images accordingly. Finally we added the findings from the prior two sections together and input the muscle force data for the purpose of computing the final results using FE modeling.

### Participant

An adult male of Chinese descent was recruited for this investigation (age: 26 years; body height: 185 cm; body mass: 82 kg) (forefoot strike patterns). The participant had sports three times a week at least for one hour at a time. There were no known medical conditions affecting the participant's lower limbs, and he did not suffer from any surgical injuries in the twelve months before the experiment that may have affected the findings. After being briefed on the nature of the research and its aims, the participant signed a written informed consent form. This research was granted approval by the Ningbo University Ethics Committee (protocol code: RAGH 20220810; approval date: 10 August 2022).

### Shoes

In this research, the individual characteristics of each participant's feet were collected using a foot-scanning machine (VAS-39, Orthobaltic, Lithuania) and a 3D printer (Dragon(L) 3D Printer, Winbo, China). A plastic foot model was built using data obtained from a foot scanner. Ningbo Jiangbei Feibu Sporting Products Co., Ltd., of Ningbo, China, utilized the scanned data to construct a shoe tree, after which the final bionic shoes were manufactured. Figure 1 depicts the processes used to create BS.

### Biomechanic Variables’ Collection and Processing

A biomechanic lab (Ningbo University's Research Academy of Grand Health) served as the setting for all tests. A force plate and an eight-camera Vicon motion capture system (Oxford Metrics Ltd., Oxford, UK) were used to gather data on dynamics and kinematics (Kistler, Switzerland). Kinematic and dynamic data were collected at 200 Hz and 1000 Hz, respectively. At a frequency of 1000 Hz, electromyography (EMG) equipment (Delsys, Boston, MA, USA) was utilized to record activations and forces from the soleus, gastrocnemius, peroneus longus, peroneus brevis and tibialis anterior muscles (So et al., 2022; Zhou et al., 2022). Data were concurrently gathered from each device. The location of 39 markers is shown in Figure 1A.

**Figure 2:**
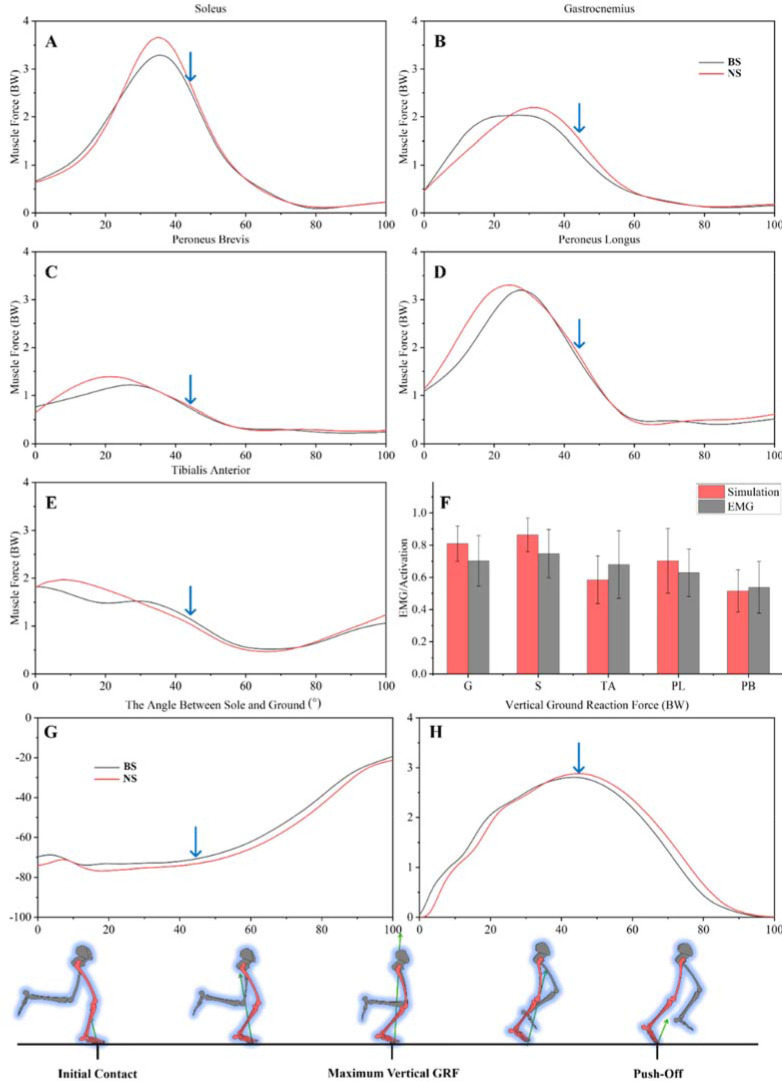
A–E: Illustration of each muscle force (blue arrow: the value to take into the model); F: Illustration of EMG/activation of each muscle. The scale on the left of the image shows that 0 (no activity) ~ 1 (full activity)**;** G: The angle between the sole and the ground; H: Illustration of the vertical GRF.

The participant ran along a 10-m track at his own pace to gather dynamic data (Figure 1B). First contact was defined as occurring when the GRF exceeded 10 N (Xu et al., 2023; Zhou et al., 2022). The participant recorded ten data trials at a self-selected running speed. For additional data analysis, an average of the ten trials was calculated. The boundary conditions of the finite element model were determined by incorporating the average value of ten collected data sets. Muscle activation datasets that were taken from the EMG signal were used to create muscular force datasets. There were no discernible changes between the EMG data and musculoskeletal models when comparing the muscle activation (Figure 2F).

OpenSim was used to explore and calculate biomechanical variables (Stanford University, Stanford, CA, USA). In a previous study, the techniques were used to gather information on muscle force and activation output (Zhou et al., 2022). The data from the running phases were retrieved, and the data from the stance phase was stretched into a time series curve with 101 data points using a customized MATLAB script. By employing this approach, data pertaining to a running stance phase can be consolidated into a coherent dataset, and subsequently standardized to a unitary scale ranging from 0 to 101. This ensured that the precise value of vertical GRF and muscular force were integrated into the FE model.

We incorporated peak vertical GRF into the FE model, exported muscle force data from the same time node into the model, and used the angle between the sole and the ground to determine the position and the angle of the FE model, as shown in Figure 2G and H (contact angle and vertical GRF) and Figure 2 A–E (muscle force).

### FE Analysis

While wearing BS and with the foot positioned at the specified angle (sole and ground angle), CT and MRI images of the participant's right foot were acquired at 2-mm intervals. Mimics 21.0 (Materialise, Leuven, Belgium) was used to segment the two-dimensional image, and Geomagic Studio 2021 was used to create and fine-tune a three-dimensional model of the bone, ligaments, Achilles tendon, bulk soft tissue, and bionic shoes (Geomagic, Inc., Research Triangle Park, NC, United States). The components were imported into SolidWorks 2017 when they were ready to be turned into solids (SolidWorks Corporation, Waltham, MA, United States). By building a solid between the contacting surfaces of two bones, the structure of cartilage was modeled. In order to simulate NS, the BS outsole was removed using SolidWorks, and then material of the same thickness was added on the top of the midsole.

Both two models' contacts were meshed and set up using Workbench 2021 (ANSYS, Inc., Canonsburg, PA, USA). Tetrahedral meshes were used to disintegrate every solid. Based on the age matching model, which had already passed the mesh convergence test, the mesh sizes of the bulk soft tissue, bone, shoes, and cartilage were modified at 3 mm, 2 mm, 2 mm, and 0.5 mm, respectively. Moreover, local refining was carried out while taking into consideration the geometry of the contact zone. Workbench provided automatic contact detection for components. Using an algorithm based on surface proximity, we produced possible contact pairings. Face-to-face contact served as a simulation of the interface between the bone's surface and the cartilage. The surface of the bone made frictionless contact with the cartilage (Athanasiou et al., 1998). All the bones and cartilage were anchored to the soft tissue that was encapsulated. To simulate the interaction between the foot, the shoes, and the ground, a contact surface with a friction coefficient of 0.6 was utilized (Yu et al., 2013). Each component of the shoes was set to bind, as well as the remaining structures.

### Boundary and Loading Condition

**Figure 3:**
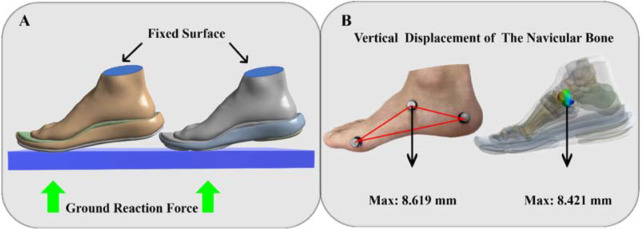
A: Illustration of fixed and loading condition; B: Vertical displacement validation of the model.

The vertical GRF (Figure 2H) from the gathered data was used to compute the force value of peak vertical GRF, which was applied evenly to the ground. The interface between the tibia and fibula was bound (Figure 3A). Five muscles were added to this model's muscular connection sites (Figure 2A–E) (Chen et al., 2012).

With the exception of the encapsulated soft tissue, all materials were thought to be isotropic and linear elastic materials, and their properties were derived from past research (Gefen et al., 2000; Gu et al., 2010; Pailler-Mattei et al., 2008; Wu, 2007). Two material constants, Young's modulus (E) and Poisson's ratio (v), were selected to represent elasticity. The enclosed soft tissue was described as a nonlinear hyperelastic material using the Moonley Rivlin model. The material properties of each component are listed in Table 1.

**Table 1:** Material properties of the components in the finite element model.

Component	Elastic modules (MPa)	Poisson's ratio v
Bulk soft tissue	Second-order polynomial strain hyperelastic model (C_10_ = 0.8556, C_01_ = 0.05841,C_20_ = 0.03900, C_11_ = 0.02319, C_02_ = 0.00851, D_1_ = 3.65273)	/
Bone	7300	0.3
Cartilage	1	0.4
Ligament	260	0.4
Plantar Fascia	350	0.4
Achilles Tendon	816	0.3
InSole	1.98	0.35
Mid-Sole	2.49	0.35
Out Sole	3.85	0.4
Plate	17000	0.4

### Validation of FE Models

In order to validate the FE foot model, the forefoot running state was simulated and compared to the experimentally recorded deformation of the navicular bone. In a clinical sense, the navicular bone's displacement serves as a proxy for the foot deformation index. In manual measurements, the node at the tuberosity of the navicular bone on the medial side is often utilized as the reference point. Calculating the vertical displacement from this node while the whole-body weight is being supported. The comparison between the measured navicular deformation (Picciano et al., 1993) and the FE stimulated result is shown in Figure 3B.

### Bone Stress Distribution Feature Classification and Recognition

A total of 10 bones were selected for stress distribution feature classification and recognition: the first to the fifth metatarsal (MT1, MT2, MT3, MT4, MT5), and the first to the fifth proximal phalanx (PP) (PP1, PP 2, PP3, PP4, PP5). Each bone stress data were divided into five cases: 1) stress values corresponding to all the nodes; 2) stress values corresponding to first 50% of nodes; 3) stress values corresponding to first 20% of nodes; 4) stress values corresponding to first 10% of nodes; 5) stress values corresponding to first 5% of nodes. A total of 50 (5 cases in 10 bones) data sets were substituted into the classification and recognition algorithm model. This study selected the K-nearest neighbor algorithm (KNN), the support vector machine (SVM), and the artificial neural network (ANN) as the feature recognition and classification model (Cover and Hart, 1967; Xu et al., 2022b).

For the KNN, the Euclidean distance k was set to 5 (Cover and Hart, 1967; Xu et al., 2022a). For the SVM, the linear kernel function was used to turn the input feature's data into a higher-dimensional space, the soft margin idea was used to cope with the possibility of misclassifications, and the regularization constant C was set to 1 (Cortes and Vapnik, 1995; Xu et al., 2022b). For the ANN, the input layer, the hidden layer, and the output layer were all set to one in this study, the batch size was set to 25, and the max epoch was set to 1000. The Sigmoid type activation function was used to get the neural network output (Xu et al., 2022a, 2022b). The node of the input layer was determined according to the number of input features, the node of the hidden layer was determined according to the group number of input data, and the node of the output layer was determined based on the number of classes (Kohonen, 1988). The 10-fold cross-validation was used in all classification models.

## Results

### Stress Distribution

**Figure 4:**
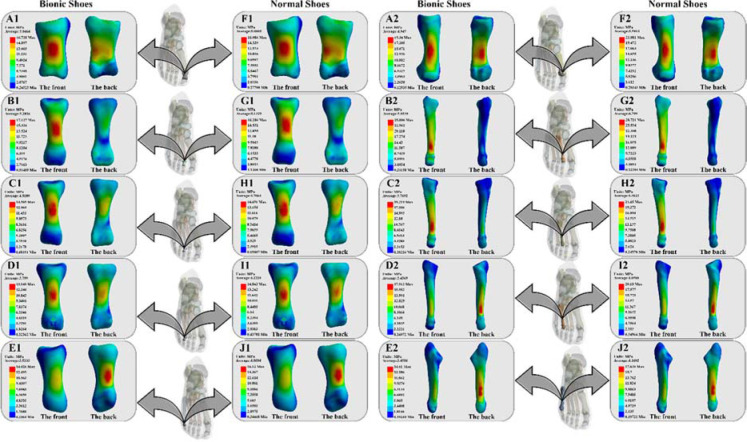
Illustration of the stress distribution of the 1^st^–5^th^ proximal phalanx and metatarsal bones between BS and NS during forefoot running.

In Figure 4, the BS and NS stress distribution on the 1^st^–5^th^ proximal phalanx bones during forefoot running is reported. The average (BS: 5.05 MPa; NS: 5.00 MPa) and maximum (BS: 16.73 MPa; NS: 16.09 MPa) stress values of the PP1 bone are displayed in Figures 4A1 and 4F1. The average (BS: 5.28 MPa; NS: 5.13 MPa) and maximum (BS: 17.13 MPa; NS: 16.21 MPa) stress values of the PP2 bone are shown in Figures 4B1 and 4G1. Figures 4C1 and 4H1 show the average (BS: 4.52 MPa; NS: 4.71 MPa) and maximum (BS: 14.51 MPa; NS: 14.69 MPa) stress values of the PP3 bone. In Figures 4D1 and 4I1, the average (BS: 3.80 MPa; NS: 4.22 MPa) and maximum (BS: 13.85 MPa; NS: 14.84 MPa) stress values of the PP4 bone are reported. The average (BS: 3.52 MPa; NS: 4.06 MPa) and maximum (BS: 14.03 MPa; NS: 16.12 MPa) stress values of the PP5 bone are displayed in Figures 4E1 and 4J1.

In Figure 4, the BS and NS stress distribution on the 1^st^–5^th^ metatarsal bones during forefoot running are reported. Figures 4A2 and 4F2 display the average (BS: 4.95 MPa; NS: 5.59 MPa) and maximum (BS: 19.34 MPa; NS: 21.88 MPa) stress values of the MT1 bone. Figures 4B2 and 4G2 show the average (BS: 5.95 MPa; NS: 6.80 MPa) and maximum (BS: 25.81 MPa; NS: 28.72 MPa) stress values of the MT2 bone. The average (BS: 3.77 MPa; NS: 4.36 MPa) and maximum (BS: 19.22 MPa; NS: 21.65 MPa) stress values of the MT3 bone are presented in Figures 4C2 and 4H2. In Figures 4D2 and 4I2, the average (BS: 3.46 MPa; NS: 4.05 MPa) and maximum (BS: 17.91 MPa; NS: 20.18 MPa) stress values of the MT4 bone are shown, while Figures 4E2 and 4J2 display the average (BS: 3.46 MPa; NS: 4.16 MPa) and maximum (BS: 14.81 MPa; NS: 17.64 MPa) stress values of the MT5 bone.

### Bone Stress Distribution Feature Classification and Recognition

**Figure 5:**
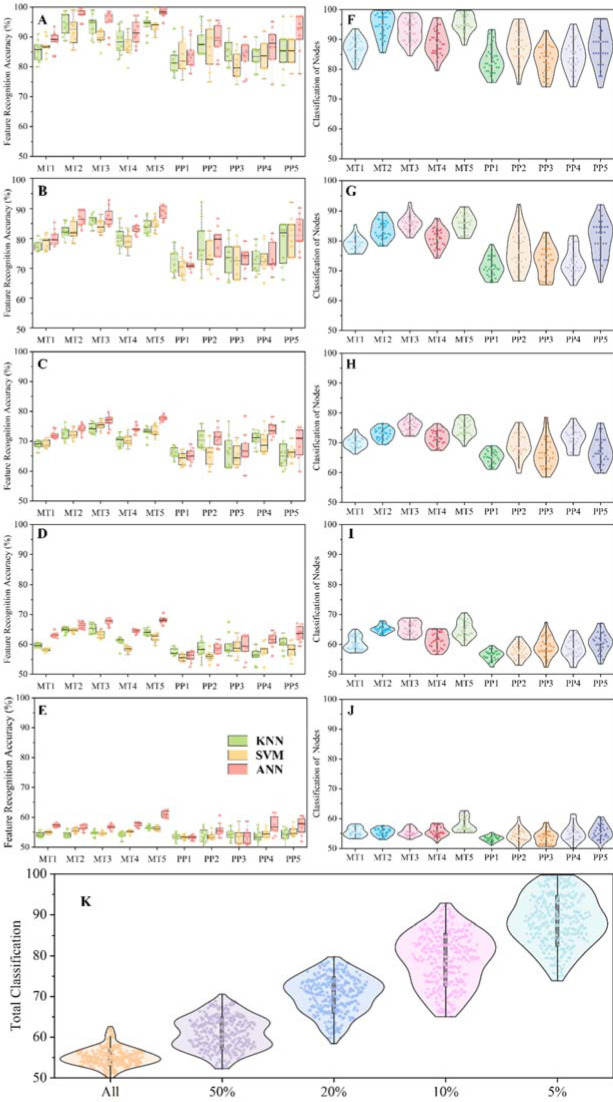
A–E: Results of the three different classification algorithm models between BS and NS during forefoot running, including the features classification and recognition accuracy rate in each contrasting scenario. F–J: Results of the total classification algorithm models between BS and NS during forefoot running, including the features classification and recognition accuracy rate in each foot bone. A and F: First 5% of nodes; B and G: First 10% of nodes; C and D: First 20% of nodes; D and I: First 50% of nodes; E and J: All nodes. K: Total classification and recognition accuracy of all features in the different nodes of MT and PP bones.

The bone stress of the three different classification algorithm models with regard to the classification of features and the recognition accuracy rate in each contrasting situation are reported in Figure 5A–J. Among them, the bone stress classification of features and the recognition accuracy rate of MT2, MT3 and MT5 are higher than other foot bones in first 5%, 10%, 20% and 50% of nodes.

The total classification and recognition accuracy of all features in the different nodes of MT and PP bones are reported in Figure 5K. Starting from all nodes to first 5% of nodes, it can be seen that all classification and recognition accuracy of a total of five different nodes show a growth pattern, and the fewer the number of nodes the higher the classification and the recognition accuracy rate.

## Discussion

The purpose of this study was to compare and analyze the differences between BS and NS forefoot strike patterns of running using the FE method. As part of our investigation, we separated the forefoot area when forefoot running as a way to create a small and independent area of instability. We hypothesized that the foot bone stress value would be lower when running on the forefoot with BS than when running with NS. More specifically, we further hypothesized that the metatarsal bones stress value would be lower when running on the forefoot with BS than when running with NS.

From Figure 5, we observed that the classification and recognition accuracy of MT2, 3 and 5 starts from first 50% of nodes, and the lower the number of nodes, the higher the classification and recognition accuracy of these three bones. Moreover, it was found that a 100% classification and recognition accuracy rate at first 5% of nodes were also observed. This method of analysis seems reasonable in terms of foot structure (Huson, 1991). The vertical distance between MT5 and the ground when running on the forefoot is the closest, thus the stress classification and recognition accuracy rate to which MT5 is subjected is also relatively high. The reason for the high classification and recognition accuracy rate of MT2 and MT3 may be due to the fact that the unstable condition involved more muscles in the movement, leaving the entire foot and ankle in a neutral position, thus increasing the MT2 and MT3 stress values. Therefore, through bone stress distribution feature classification and recognition, it can be further explained that these three metatarsal bones play a crucial role in the overall forefoot running process.

Metatarsal stress fractures account for around 10–20% of all stress fractures in athletes and are notably prevalent in runners (Matheson et al., 1987). Among forefoot runners, the probability of such metatarsal stress fractures risks will be higher than in rearfoot runners (Kernozek et al., 2014). It has been demonstrated that the magnitude of metatarsal stress values is an important indicator in the evaluation of metatarsal stress fractures (Madjarevic et al., 2009). Comparing the results of our study, it can be seen in Figure 4 that the metatarsal stress values in forefoot strike patterns with BS were less than with NS. This also suggests that although forefoot strike patterns increase the risk of metatarsal stress fractures, the risk of metatarsal stress values fractures can be effectively reduced through the use of BS. When comparing with previous studies on unstable shoes (Lohrer et al., 2008), we found that this is most likely due to the BS increasing instability and passively activating the lower limb function to improve it as a way to reduce metatarsal stress values.

Hallux valgus is a common orthopedic disorder characterized by a deformation and dysfunction of the great toe (Chen et al., 2001). Previous studies have shown that lower PP1 and MT1 stress values during a gait cycle can effectively reduce the probability of hallux valgus (Gu et al., 2014). Our results show that the stress values in PP1 and MP1 are less when running with BS forefoot strike patterns than when using NS, which is in agreement with previous studies. This would also suggest that BS might be able to reduce the probability of hallux valgus injury. It has been indicated that BS may have a positive effect on the prevention of hallux valgus injury in adolescent children aged 12–14 years (Klein et al., 2009). Additionally, stress value fractures of MT5 are also common in metatarsal injuries (Torg et al., 1984). Previous studies have shown that lower MT5 stress value is effective in reducing the incidence of MT5 fractures (Madjarevic et al., 2009).

Our results show that the stress value on MT5 is less when running with BS forefoot than when using NS. This also proves that perhaps BS forefoot running can also reduce the probability of MT5 fractures.

The above analysis and inferences for BS prove that running with BS forefoot strike patterns is better than when using NS, but what exactly causes this? From the structure of the ankle joint and the forefoot strike patterns, forefoot running is a way of running with the forefoot as the only contact with the ground. The instability of the ankle joint when running on the forefoot causes the lower limb muscles to be more involved in the gait as a way to ensure a reduction in the risk of ankle injury. Although this is also the case with the NS forefoot run, NS lack an instability factor compared to the BS forefoot run. The instability of the sole structure makes BS running an additional factor. When running with a BS forefoot strike pattern, the instability of the sole and the instability of the ankle joint combine to create an additional instability factor. We further speculate that this may be due to the fact that the two instabilities cause the foot and lower extremity muscles to be more involved in the movement, resulting in lower skeletal stress in the foot. In addition, we believe that this concentration of the entire foot function in the forefoot may also be one of the reasons.

We must acknowledge that the current study has some limitations. Firstly, only one healthy male participant was chosen for this study. Since individuals differ, the study's findings could have different final conclusions with another sample. Secondly, it was decided to divide the bone into a cortical layer and a cancellous layer during FE modeling. The stress value of the bone would increase if it were considered to be a linear elastic material, which would necessitate further simplifications of some secondary organization and structure of the complex body and thus would not be entirely accurate. Furthermore, static structure analysis does not accurately depict the entire running stance phase. Explicit dynamics analysis utilizing a larger sample size is required for future work.

## Conclusions

In summary, this study investigated and analyzed differences between BS and NS forefoot strike patterns of running using the FE method. The results of this paper indicate that BS forefoot running helped reduce the probability of the occurrence of metatarsal stress fractures in the present participant. In addition, the findings further revealed that BS may have important implications for the prevention of hallux valgus, which may be more effective in adolescent children. Finally, this study presents a post-processing method for FE results, which is of great significance for further understanding and exploration of FE results.
